# HIV epidemic among Brazilian women who have sex with women: An ecological study

**DOI:** 10.3389/fpubh.2022.926560

**Published:** 2022-08-03

**Authors:** Ana Luisa Lemos Bezerra, William Sorensen, Taymara Barbosa Rodrigues, Sara Melissa Lago Sousa, Márcia Simão Carneiro, Sandra Helena Isse Polaro, Aline Maria Pereira Cruz Ramos, Glenda Roberta Oliveira Naiff Ferreira, Elucir Gir, Renata Karina Reis, Eliã Pinheiro Botelho

**Affiliations:** ^1^Nursing Graduate Program, Federal University of Para, Belém, Brazil; ^2^Health & Kinesiology Department, University of Texas at Tyler, Tyler, TX, United States; ^3^College of Nursing, Universidade de São Paulo, Ribeirão Preto, Brazil

**Keywords:** HIV, women who have sex with women, health policies, Brazil, temporal series analysis

## Abstract

The influences of public policies fighting HIV among women who have sex with women is scarcely studied. This study aimed to analyse the time series of human immunodeficiency virus (HIV) epidemic, between 2007 and 2020, among Brazilian women who have sex with women, in order to evaluate the effect of Brazilian policies for fighting HIV in this subpopulation compared to women who have sex with men (WSM). This ecological study employed HIV and acquired immunodeficiency syndrome (AIDS) new cases among women who have sex strictly with women (WSW), women who have sex with men and women (WSMW), and WSM reported to the Sistema de Informação de Agravos de Notificação from 2007 to 2020. Crude Brazilian and regional annual age-adjusted HIV/AIDS population-level incidence rates were calculated for WSW, WSMW and WSM. The rates were then analyzed using the Joinpoint regression model. A total of 102,890, 757, and 1,699 notifications of WSW, WSMW, and WSM living with HIV/AIDS were reported during the study period, respectively. South Brazilian region had the greatest HIV/AIDS incidence rates among WSM and bisexual women while the North region had the greatest incidence among WSW. In the WSM population, the temporal trends showed at least one stable or an increasing trend period from 2007 to 2013 or 2014, followed by one decreasing trend in all Brazilian regions. While among the WSMW most of the regions had a stable trend period from 2007 to 2020, in WSW group most of the trends had only one decreasing period. The decreasing trends were faster in WSM than in WSW. These results suggest a low efficiency of Brazilian policies for fighting HIV among WSW and WSMW and show the necessity of implementing new policies specific to this population.

## Introduction

Approximately 37.7 million people worldwide live with human immunodeficiency virus (HIV) and 53% of these people are women (1). However, in the last decade, the number of new HIV infections among women decreased by 27% (2). Among Brazilian women, a total of 346,791 HIV/AIDS cases were reported until 2020, with a decrease of 50% in the incidence rate between 2010 and 2020 (2010: 16; 2020: 8; 100,000 inhabitants). However, regional discrepancies exist, with the South region of Brazil having a high proportion of women living with HIV, followed by the North and Northeast, Southeast and Midwest regions (3).

The HIV epidemic among women who have sex with women (WSW) and women who have sex with men and women (WSMW) is unknown in Brazil. In the history of the HIV epidemic, WSW and WSMW have been occluded by a heterosexist exposure group due to their lower risk to HIV or other sexually transmitted infections (STI), and their relatively less frequent counts. However, they are not free of STI risks. Cases of HIV have been reported among WSW in the U.S. and Africa. HIV and other STI can be transmitted from women to women by sharing toys or different kinds of exposed fluid during sex (4). A study among Brazilian WSW showed a human papillomavirus (HPV) prevalence of 45.3%, 2.0% *Chlamydia trachomatis*, 0.7% HIV, and 1.3% *gonorrhea*, trichomoniasis, and syphilis (5).

Multiple sexual partnerships, inconsistent use of sex contraceptives, forced sexual intercourse with men, social prejudice and stigma and not receiving sexual health education from health professionals have been associated with the risk of STI among WSW and WSMW (5). In addition, the myth that WSW are protected against STI contributes further to their risk of STI (4, 6). The international guidelines explicitly indicate, as preventative forms against STI in WSW, the use of barriers to reduce contact with body fluids (condom, gloves, latex sheet and dental dam) and treatment for genital lesions (7). However, Brazilian WSW were less likely to use sex barrier protection and attend gynecologic consultation than bisexual women (8).

In 2011, the Brazilian Ministry of Health implemented a National Health Policy for lesbians, gays, bisexuals, and transgenders (LGBT) within the scope of the Unified Health System (SUS). The main objective of this policy was to promote integral health assistance for the LGBT population, eliminating discrimination and institutional prejudice (9). However, most of the Brazilian policies and health campaigns to combat HIV are exclusive to heterosexual women (WSM). For example, in 2012, the Ministry of Health implemented HIV tests as a prenatal routine. Other public policies to combat HIV were generally for the public, such as the policy Treatment as Prevention, implemented in 2013, in which people diagnosed with HIV receive antiretroviral therapy regardless of T-CD4 cell count. Another example is decentralization of HIV tests in primary health care locations, in 2015, with the main goal of increasing test coverage (10). Regarding female sexual minorities, it was only in 2016 that the Brazilian Ministry of Health included transgender women in the health campaigns (11).

However, even today none is known about how these implemented policies are influencing the HIV epidemic among WSW and WSMW in Brazil. Therefore, this study's main goal was to analyse temporally the HIV/AIDS incidence rates among WSW, WSMW and women who have sex with men (WSM) from 2007 to 2020, comparing the results among the three groups. Temporal analysis techniques are useful tools to provide a better understanding about how the HIV epidemic behavior over the time and to evaluate the efficiencies of the public policies designed to eliminate the virus.

## Methods

This study is an ecological study.

### Study design and population

**Figure 1:**
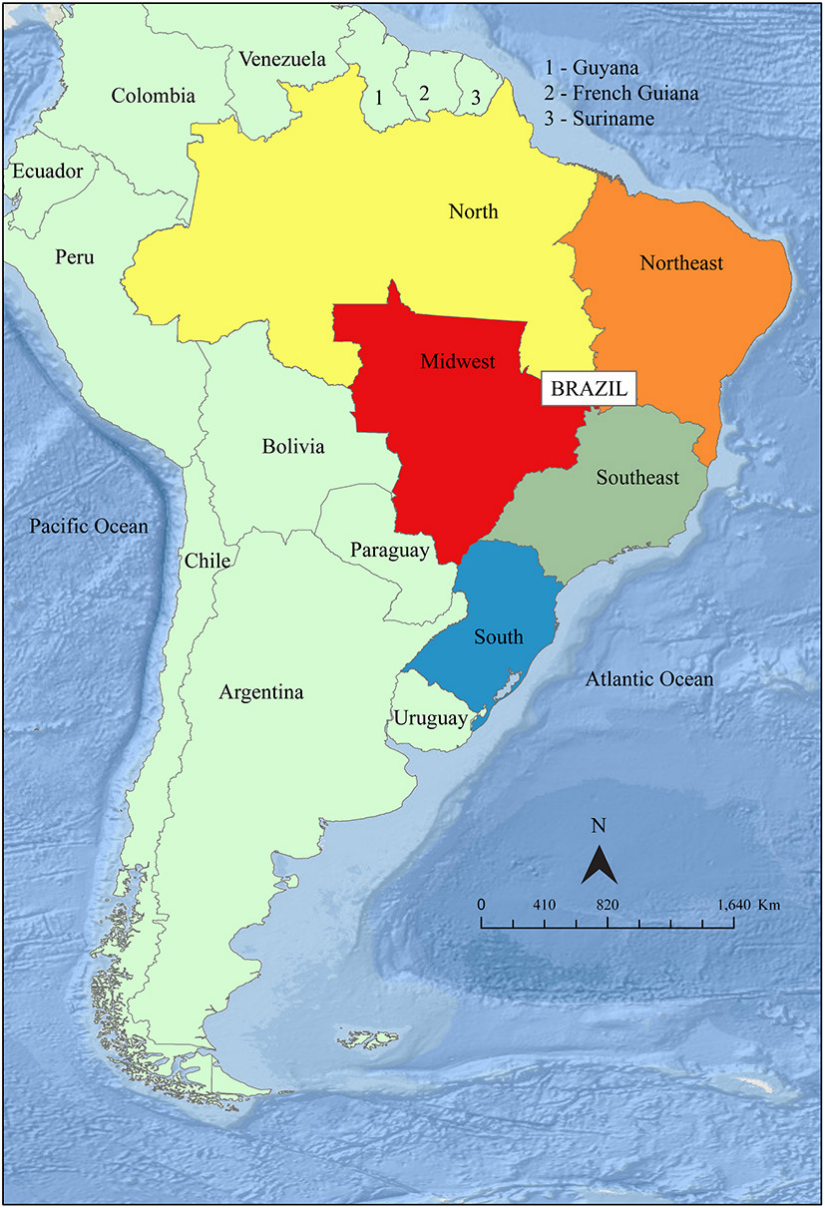
Map of Brazil showing the five Brazilian regions. The map was constructed by the authors.

Brazil is in the South American continent with a land area of 8,510,345,538 km^2^ divided into 26 states, 1 federal district, 5,568 municipalities and an estimated population of 86,883,835 women aged 15 years and over. Brazil's territory is divided into five regions: North, Northeast, Midwest, Southeast and South (Figure 1). Brazil is rated 84th in the Human Development Index (HDI = 0.765) among other countries (12). Brazil has a great regional disparity of HDI and SUS outcomes, with the North region having the lowest HDI of 0.719 and the Southeast region having the lowest primary healthcare coverage of 54.6% (13).

The study population was composed of all cases of HIV/AIDS in WSM, WSMW, and WSW aged 15 years and over and reported to the Sistema de Informação de Agravos de Notificação. Only reports of women residing in Brazil were included.

### Variables

All variables were obtained from the website of the Departamento de Informática do Sistema Único de Saúde (DATASUS), an agency responsible for providing to SUS information and informatics support to help Brazilian health authorities plan and execute actions to improve the health of the population.

The following variables were collected: year of notification, sexual orientation, age, schooling level, skin color/race, Brazilian region of residency, and annual projected population of women aged 15 years and over. Data were grouped by region, quality-checked, and all inconsistencies were removed.

The regional and Brazilian annual HIV/AIDS incidence rates in WSM, WSMW and WSW were calculated by dividing the number of HIV/AIDS reports in each specific category by the yearly regional and Brazilian projected female population in the specific age range. The results were then multiplied per 100,000 inhabitants.

### Data analysis

Descriptive analysis was conducted using Microsoft Office Excel 365 2019 (Microsoft Corporation, Santa Rosa, CA, USA) and the results were expressed as absolute (*n*) and relative frequencies (%).

The Joinpoint regression model was adopted for temporal analysis by using the Joinpoint Trend Analysis software 4.8.0.1 (National Cancer Institute, Calverton, MD. USA). In this regression model, joinpoints are fitted in a linear regression until the joints distinguish two trend periods. In this analysis, the regional rates were directly age-adjusted following the Joinpoint regression model to avoid population variation influences. The best-fitting Joinpoint regression model was accessed by the Monte Carlo permutation test, which employed 4,999 permutations. The annual percentage change (APC), 95% confidence interval (95% CI), and *p*-value were considered for each category. Trends were considered increasing or decreasing if the APC was positive or negative, respectively, and *p*-value <0.05. Otherwise, they were deemed stationary.

### Ethics approval

According to the Brazilian Heath Council, Resolution no. 510, year 2016, studies employing non-personally identifiable public domain data do not require approval from the Ethics Committee. All data are publicly available on DATASUS website.

## Results

**Figure 2:**
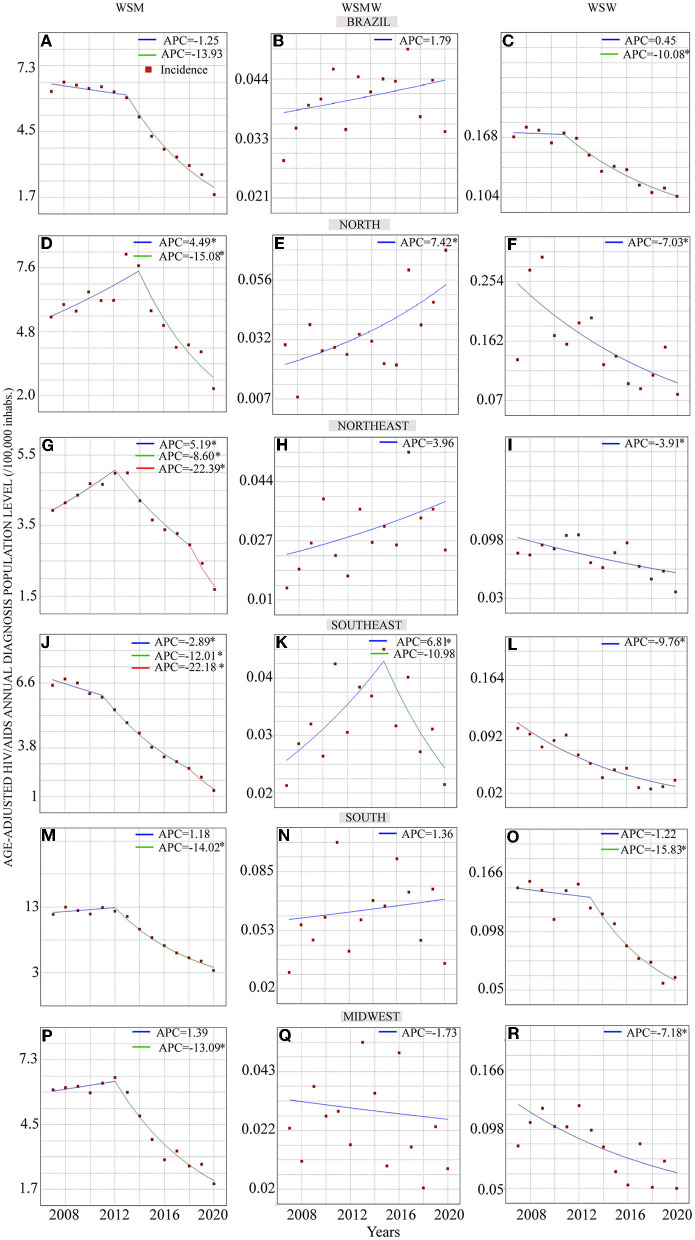
Temporal trend analysis of HIV/AIDS regional aged-adjusted incidence rates in Brazilian WSM, WSMW and WSW. **(A–C)** The trends of the crude HIV/AIDS incidence rates in Brazil for WSM, WSMW, and WSW, respectively. The Brazilian regional aged-adjusted trends for WSM are shown in figures for **(D,G,J,M,P)**, and in figures **(E,H,K,N,Q)** for WSMW women, and for WSW in figures **(F,I,L,O,R)**. The blue, green and red traces represent a trend period. The red squares show the noticed HIV/AIDS incidences rates. *p-value <0.05.

During the study period, 102,890, 757, and 1,699 new WSM, WSMW and WSW living with HIV/AIDS. For the 13 years of the study duration, the HIV/AIDS aged-adjusted population-level diagnosis rates among WSM, WSMW and WSW were higher in the South and North regions, respectively. Figures 2A,D,G,J,M,P; Table 1 show the results of temporal trend analysis of crude Brazilian and regional age-adjusted population HIV/AIDS incidence rate among WSM. In Brazil (Figure 2A), in the South (Figure 2M) and Midwest (Figure 2P) regions, a stable period was observed, followed by a decreasing trend period. In the North (Figure 2D) and Northeast regions (Figure 2G), an upward trend followed by one and two downward periods were found, respectively. Three downward trends were observed in the Southeast region (Figure 2J), with the third being faster than the previous ones. Considering average APC (AAPC), the North and Southeast regions had slow (−5.1) and fast (−11.0) downward trends, respectively.

**Table 1:** Temporal trend analysis of annual aged-adjusted HIV/AIDS population-level incidence rates in Brazilian WSM, 2007–2020.

**Region**	**Time period**	**APC**	**95% CI**	***p*-Value**	**AAPC**	**95% CI**	***p*-value**
Brazil	2007–2013	−1.25	−3.6; 1.2	0.24	−8.3	−9.7; −6.9	<0.001
	2013–2020	−13.93	−16.1; −11.7	<0.001			
North	2007–2014	4.49	0.0; 9.2	0.053	−5.1	−8.2; −1.8	0.003
	2014–2020	−15.08	−20.5; −9.3	<0.001			
Northeast	2007–2012	5.19	2.0; 8.5	0.006	−6.0	−8.5; −3.3	<0.001
	2012–2018	−8.70	−11.5; −5.9	<0.001			
	2018–2020	−22.4	−35.7; −6.3	0.015			
Southeast	2007–2011	−2.90	−6.1; 0.3	0.068	−11.0	−13.3; −8.6	<0.001
	2011–2018	−12.01	−13.9; −10.0	<0.001			
	2018–2020	−22.18	−35.7; −5.7	0.018			
South	2007–2012	1.18	−1.6; 4.1	0.423	−8.5	−9.7; −7.2	<0.001
	2012–2020	−14.02	−15.6; −12.4	<0.001			
Midwest	2007–2012	1.39	−3.9; 7.0	0.602	−7.8	−10.0; −5.5	<0.001
	2012–2020	−13.09	−15.9; −10.2	<0.001			

*APC, annual percent change; 95% CI, 95% confidence interval; AAPC, annual average percentual change*.

Figures 2B,E,H,K,N,Q; Table 2 shows the temporal analysis results for WSMW. Only in North and Southeast regions (Figures 2E,K, respectively) there was an upward trend. However, while in the North region there a continuous increase trend, in the Southeast region the upward trend was noticed only between 2007 and 2015 followed by a stable period from 2015 to 2020. In Brazil and in the other Brazilian regions the time series were considered stable.

**Table 2:** Temporal trend analysis of annual aged-adjusted HIV/AIDS population-level incidence rates in Brazilian WSMW, 2007–2020.

**Region**	**Period**	**APC**	**95% CI**	***p*-Value**	**AAPC**	**95% CI**	***p*-Value**
Brazil	2007–2020	1.79	−1.5; 5.2	0.26	—	—	—
North	2007–2020	7.4	2.3; 12.9	0.008	—	—	—
Northeast	2007–2020	4.0	−1.0; 9.2	0.11	—	—	—
Southeast	2007–2015	6.81	0.1;−14.0	0.048	−0.4	−5.9; 5.4	0.885
	2015–2018	−11.0	−22.1; 1.8	0.59			
South	2007–2020	1.36	−3.9; 6.9	0.59	—	—	—
Midwest	2007–2020	−1.73	−1.5; 5.2	0.26	—	—	—

*APC, annual percent change; 95% CI, 95% confidence interval; AAPC, annual average percentual change*.

Figures 2C,F,I,L,O,R; Table 3 show the temporal trend analysis results of HIV/AIDS age-adjusted population incidence level rates among WSW. Only in Brazil (Figure 2C) and in the South region (Figure 2O) did the time series present two trend periods, a stable period followed by a downward one. The other areas had only one downward trend. Furthermore, comparison of the APC between WSM and WSW showed that the time series among WSM had a higher decreasing speed in the second trend period than that among WSW in Brazil and the North, Northeast, Southeast, and Midwest regions (Brazil – HTW: APC = −13.93, WSW: APC = −10.08; North – HTW: APC = −15.08, WSW: APC = −7.03; Northeast – HTW: APC = −8.60, WSW: APC = −3.91; Southeast – HTW: APC = −12.01; WSW: APC= −9.76; Midwest – HTW: APC = −13.09; WSW: APC = −7.18). Only in the South region of Brazil was the APC faster among WSW than among WSM (−15.82 vs. −14.02, respectively). The slower trend period decreasing among WSW was noticed in the Northeast region (APC = −3.91) and the faster one was found in the South region (2013–2020: APC = −15.09).

**Table 3:** Temporal trend analysis of annual aged-adjusted HIV/AIDS population-level incidence rates in Brazilian WSW, 2007–2020.

**Region**	**Time period**	**APC**	**95% CI**	***p*-value**	**AAPC**	**95% CI**	***p*-value**
Brazil	2007–2013	−1.25	−3.6; 1.2	0.24	−8.3	−9.7; −6.9	<0.001
	2013–2020	−13.93	−16.1; −11.7	<0.001			
North	2007–2014	4.49	0.0; 9.2	0.053	−5.1	−8.2; −1.8	0.003
	2014–2020	−15.08	−20.5; −9.3	<0.001			
Northeast	2007–2012	5.19	2.0; 8.5	0.006	−6.0	−8.5; −3.3	<0.001
	2012–2018	−8.70	−11.5; −5.9	<0.001			
	2018–2020	−22.4	−35.7; −6.3	0.015			
Southeast	2007–2011	−2.90	−6.1; 0.3	0.068	−11.0	−13.3; −8.6	<0.001
	2011–2018	−12.01	−13.9; −10.0	<0.001			
	2018–2020	−22.18	−35.7; −5.7	0.018			
South	2007–2012	1.18	−1.6; 4.1	0.423	−8.5	-9.7; -7.2	<0.001
	2012–2020	−14.02	−15.6; −12.4	<0.001			
Midwest	2007–2012	1.39	−3.9; 7.0	0.602	−7.8	−10.0; −5.5	<0.001
	2012–2020	−13.09	−15.9; −10.2	<0.001			

*Brazil, 2022. APC, annual percent change; 95% CI, 95% confidence interval; AAPC, annual average percentual change*.

## Discussion

This study is the first to show the temporal scenario of the HIV epidemic among WSMW and WSW in Brazil. The results showed that the HIV/AIDS incidence rates were much higher among WSM than in WSMW and WSW. The Southeast region had the greatest HIV/AIDS incidence rates among WSM and WSMW, while for the WSW it was noticed in the North region. While in WSMW the results shown a stable trend of the HIV/AIDS incidence in the study period in almost the Brazilian regions, in WSM and WSW there was a downward trend. WSW had slower decreasing trends than WSM. The Northeast region had the slower HIV/AIDS incidence rates decreasing among WSW and the South region had the faster one. In addition, at least two trend periods were found for WSM in all regions, such a period was observed only in Brazil and in the South region for WSW.

Given that this study is an ecological study, causality cannot be claimed. Another limitation of this study is the issue of underreported cases. This study included the year 2020 whereby underreporting could even be higher than expected due to the displacement of professionals working during the COVID-19 pandemic (14). In addition, the quality of provided information is dependent on healthcare employees responsible for filling out the forms, raising possible misclassification bias. For example, information about transgender women is unknown since DATASUS shows only bisexual, heterosexual and homosexual categories. Furthermore, for fearing the stigma and social prejudice WSW and WSWM can omit their sexual orientation and, therefore, the number of WSW living with HIV/AIDS may be underestimated. Future studies should consider analyzing the transmission routes of HIV among WSW, the influence of social determinants of health and the effect of the COVID-19 pandemic on the HIV epidemic.

All the breakpoints noticed among WSM (2011–2013) suggested the efficiencies of policies controlling HIV in this subpopulation. For example, in 2011, the Brazilian Ministry of Health implemented the Stork Network Program to reduce infant mortality. In this policy, women are followed during the prenatal stage and their babies up to 2 years after birth. The inclusion of HIV tests in the prenatal prevention and treatment routine in 2012, are other policies contributing to this decrease among HTW (10). Furthermore, all these policies succeeded partly due to the governmental public health campaigns devoted only to HIV in HTW (11).

The greatest HIV/AIDS incidence rates and the faster-decreasing trends in WSM occurred in the southern region of Brazil. For decades, the South was the first region in Brazil with the highest HIV/AIDS incidence rate. However, from 2010 to 2020, this region had the greatest decrease in HIV/AIDS incidence (45.6%) and the greatest expansion of locations for primary healthcare coverage (9.1%) (14). This expansion of locations for coverage of primary healthcare could be the reason why the fast trend of HIV/AIDS population-level incidence decreased among WSW living in South Brazil (13).

Meanwhile, the North and Northeast regions had slow decreasing trends among WSW, with the North having a increasing trend of HIV/AIDS incidence among WSWM. In addition, the North region was the only one having an upward trend among WSMW. These regions were ranked as having the highest and second highest Brazilian violence prevalence rates against women and LGBT, respectively (15). Between 2002 and 2016, of the 15 Brazilian capitals with the greatest mortality rates due to violence among LGBT people, seven were from the Northeast region and three originated from the North region (16). In Haitian women, HIV infection was strongly correlated among those who suffered from violent intimate partners (17). A study about the association of sexual violence with sexual orientation among Americans showed that WSW had more prevalence of unwanted sexual contact and unwanted sexual experiences with no physical contact than WSM (18).

Concerning to the implemented policies fighting HIV by the Brazilian Ministry of Health, there is still regional discrepancies. For example, decentralization of healthcare services for PLWHA to the primary healthcare network still not implemented in the Northern Brazil. The geographic aspects of the North region (large territorial area, raining climate, densely forested areas) and the low socioeconomic conditions of its inhabitants are barriers to people access the preventive, diagnosis, and treatment HIV-related healthcare services. In addition, the North region has a low coverage of specialized healthcare services. In the states of Pará, for example, for its 144 municipalities there are only 07 specialized healthcare centers to attend PLWHA, 33 dispensing unities of antiretroviral treatment and only 02 dispensing unities of the Pre-Exposure Prophylaxis located in Belém, the capital of the state. A pilot study in Manaus, capital of the Amazonas state, showed that the decentralization of the healthcare services promoted a better adherence on antiretroviral treatment among PLWHA (19).

The fact of finding a higher incidence rate among WSM than in WSW and WSMW may be reflex of the greater risk to HIV among WSM women compared with the two other groups. However, the lower HIV testing among WSMW and WSW must be considered. The low accessibility of WSW to healthcare sites, and the un-trained health professionals dealing with this subpopulation are relevant aspects impacting the fighting against HIV in these category groups. In Africa, WSW declared fear of accessing health services because of stigma and social prejudice against their sexual orientation (20, 21). In Canada, the risk of STI among WSW was found to be associated with sexual stigma and sexual violence. This carries other consequences, for example, not having been tested for HIV, other STIs and not having a Pap smear exam in the last 2 years, and not receiving sexual health education from health workers (22).

The inclusion of sexual minority topics to medical, allied and public health courses in university curricula is necessary. In the U.S. and Mexico, medical students declared themselves prepared to care for LGBT people but were not comfortable with doing so (23). However, another study performed on long-term care staff showed that these professionals' training to deal with the LGBT subpopulation changed their negative attitude to a positive attitude (24).

The lower HIV/AIDS incidence rates in WSMW compared to WSW may be reflex of greater notion of risk in relation to HIV among WSMW since they have sex with men and use more frequently the preservative. Even in 2022, the WSW subpopulation may still not be aware of their risk of STI (25). Changing this situation requires the elimination of social prejudices and stigmas and the improvement of the capacity of health professionals to promote sexual health among LGBT. However, the re-emergence of conservative policies in Brazil setback several social conquests that were already attained, such as the exclusion of the terms “sexual orientation” and “genre” from the National Education Plan (26).

## Conclusions

The temporal trend analysis of HIV/AIDS population-level incidence rates among WSM had at least two trend periods, stable or increasing, followed by a decreasing trend period. However, among WSMW and WSW, the trends were characterized by only one decreasing and stable period, respectively in most Brazilian regions. Additionally, the decreasing trends among WSW were slower than those among WSM. These results suggest the necessity of reinforce Brazilian public policies controlling HIV among WSW and WSMW and the necessity to implement policies specific to this subpopulation.

## Data availability statement

The datasets presented in this study can be found in online repositories. The names of the repository/repositories and accession number(s) can be found at: https://datasus.saude.gov.br/informacoes-de-saude-tabnet/.

## Ethics statement

According to the Brazilian Heath Council, Resolution no. 510, year 2016, studies employing non-personally identifiable public domain data do not require approval from the Ethics Committee.

## Author contributions

Study design: AB, GF, and EB. Study conduct: AB and EB. Data collection: AB. Data analysis: AB, GF, AR, and SS. Data interpretation: AB, TR, GF, and EB. Manuscript drafting: WS, EG, RR, MC, SP, GF, and EB. Supervisor: EB. All authors approving final version of manuscript.

## Conflict of interest

The authors declare that the research was conducted in the absence of any commercial or financial relationships that could be construed as a potential conflict of interest.

## Publisher's note

All claims expressed in this article are solely those of the authors and do not necessarily represent those of their affiliated organizations, or those of the publisher, the editors and the reviewers. Any product that may be evaluated in this article, or claim that may be made by its manufacturer, is not guaranteed or endorsed by the publisher.
